# A High Diet Quality Is Associated with Lower Incidence of Cardiovascular Events in the Malmö Diet and Cancer Cohort

**DOI:** 10.1371/journal.pone.0071095

**Published:** 2013-08-05

**Authors:** Joanna Hlebowicz, Isabel Drake, Bo Gullberg, Emily Sonestedt, Peter Wallström, Margaretha Persson, Jan Nilsson, Bo Hedblad, Elisabet Wirfält

**Affiliations:** 1 Center for Emergency, Skåne University Hospital, Malmö, Sweden; 2 Lund University, Department of Clinical Sciences in Malmö, Sweden; 3 Department of Cardiology, Skåne University Hospital, Malmö, Sweden; INRCA, Italy

## Abstract

**Aims:**

To investigate if diet quality is related to incidence of cardiovascular (CV) events.

**Subjects and Methods:**

A diet quality index based on the 2005 Swedish Nutrition Recommendations and the Swedish Dietary Guidelines was created and included six dietary components: saturated fatty acids, polyunsaturated fatty acids, fish and shellfish, dietary fiber, fruit and vegetables, and sucrose. The index ranked 17126 participants (59% women) of the population-based Malmö Diet and Cancer cohort (Sweden) on their dietary intakes. Total index score was categorized as low, medium or high. Cox proportional hazard regression was used to model associations between index score categories and index components with risk of incident CV events, with adjustment for potential confounders. The incidence of first CV events (non-fatal or fatal myocardial infarction or ischemic stroke or death from ischemic heart disease) was monitored from baseline (1991–1996) until December 31, 2008; 703 CV events occurred in women and 1093 in men.

**Results:**

A high diet quality was associated with decreased risk of CV events when compared to a low diet quality. In multivariate analysis, the risk reduction was 32% (hazard ratio = 0.68, 95% confidence interval: 0.49–0.73) in men and 27% (hazard ratio = 0.73, 95% confidence interval: 0.59–0.91) in women. When examined separately and mutually adjusted for each other, the individual components were either not associated with CV risk or marginally decreased risks were seen.

**Conclusion:**

High quality diets in line with current recommendations may reduce the risk of CV events. This study illustrates the importance of considering a combination of dietary factors when evaluating diet-disease associations.

## Introduction

The relation between intake of single nutrients or foods and cardiovascular disease (CVD) has been studied extensively. Since foods are consumed together, and nutrients are metabolized jointly, health outcome studies could benefit from the investigation of dietary patterns. [1] There is strong evidence that dietary patterns with high intakes of vegetables, legumes, fruits, nuts, whole-grain products, and mono- and polyunsaturated rather than saturated fats (e.g., “prudent” or Mediterranean diets), offer protection against coronary heart disease (CHD) and stroke. [2]–[4] Although, the general dietary recommendations are supported by several randomized controlled trials, [5]–[10] the disappointing results of large-scale interventions can cause doubt regarding the long-term benefit of dietary changes. [11] Poor compliance also makes it difficult to estimate the potential effect of dietary changes. [12] In addition, trials are often limited to investigating the effect of a limited number of dietary changes on markers for disease (e.g. blood pressure and blood lipids) due to the practical difficulties with carrying out long-term controlled trials addressing the overall dietary pattern. The evidence for the potential health effects of dietary recommendations must therefore also rely on the results from observational studies. Dietary indices, or a *priori* dietary pattern methods, are based on current nutrition knowledge and used to evaluate overall diet quality in observational settings. [13] So far, most epidemiological studies investigating dietary patterns in line with dietary recommendations have found that they are associated with decreased risk of CVD morbidity [14]–[16] and mortality. [3], [17]–[18].

We have previously described the development of a diet quality index (DQI) in the Malmö Diet and Cancer (MDC) study, and demonstrated the usefulness of this index (DQI-SNR) when assessing diet quality based on the Swedish Nutrition Recommendations of 2005 (SNR) and the Swedish Dietary Guidelines (SDG). [19] In a methodological study investigating the effect of index scoring models on the predictive capability for mortality outcomes, a high diet quality was associated with decreased CVD mortality among men, but not women. [20] Since CVD typically occurs later in women than men, the null association among women may be due to fewer CVD deaths occurring during follow-up and thus reduced statistical power. Further, there may be certain gender differences in diagnosis and treatment of CVD affecting particularly mortality outcomes. Therefore, this study aimed to explore the association between diet quality and incidence of cardiovascular (CV) events during a mean follow-up period of 14 years, in a Swedish population-based cohort of middle-aged men and women.

## Methods

### Study Population

The MDC is a prospective population-based study designed to investigate the relationship between diet and other lifestyle factors on the risk of developing cancer, with baseline examinations between March 1991 and October 1996. [21] All men (born between 1923 and 1945, aged 45–73 years, mean age 59 years) and women (born between 1923 and 1950, aged 44–74 years, mean age 58 years) living in the city of Malmö were eligible for participation. Approximately 40% (n = 28449) of the source population (n = 74138) either joined the study spontaneously or after receiving a mailed invitation, and complete nutritional data were obtained for 28098 subjects. In order to avoid influence of disease history, dietary assessment error, or dietary changes potentially related to the study outcomes, several exclusions were made prior to analysis. Subjects with a previous cardiovascular event, identified through local or national registers (n = 826, 74% men), or reporting a diabetes diagnosis and/or identified as being treated with anti-diabetic medicine (n = 800, 50% men) were excluded. Subjects classified as energy misreporters were also excluded (n = 4871, 32% men). Energy misreporting was defined as having a ratio of energy intake to basal metabolic rate outside the 95% confidence interval (Cl) of the individual’s physical activity level. [22] Finally, subjects reporting a substantial change in their eating habits in the past (due to illness or other reason) [23] were excluded (n = 4475, 35% men). The final study population included 6940 men (41%) and 10,186 women (59%). The Ethics Committee at Lund University approved the design of the MDC study (LU 51–90). Written informed consent was obtained from all participants.

### Dietary Data

Dietary history data were obtained for the MDC study by combining a 7-day menu book (“current” diet information) and a diet history questionnaire (“usual” diet information) with a 1-hour interview. The reference period for the questionnaire was the preceding year. Studies on the reproducibility and validity of the methodology have been published previously. [24], [25] A categorical variable delineating the four seasons (i.e., winter, spring, summer, and autumn) was defined based on the date of the dietary interview. Because of a slight change in the coding routines, a categorical variable (i.e., method) was defined indicating dietary data collection before or after September 1, 1994. [26].

The construction of the diet quality index (DQI-SNR), developed to assess diet quality based on the SNR issued in 2005, has been described in a previous paper. [19] The DQI-SNR includes six components: energy percentage (E %) from saturated fatty acids (SFAs), E % from polyunsaturated fatty acids (PUFAs), intake of fish and shellfish (g/week), dietary fiber (g/MJ), fruit and vegetables (g/day), and E% from sucrose. In this study, the nutrient density variables (E %) were based on non-alcohol energy. Each index component was dichotomized using pre-defined cut-offs reflecting recommended intake levels and defined as: SFA ≤14 E%, PUFA 5–10 E%, fish and shellfish ≥300 g/week, dietary fiber 2.4–3.6 g/MJ, fruit and vegetables ≥400 g/day, and sucrose ≤10 E%. The cut-off for SFA was adjusted from the recommended ≤10 E% to ≤14 E% since only 2% of the cohort had intakes below the recommended level. [19] Each component provided one point for participant intake levels within the defined cut-offs and zero points for those with intakes above or below the recommended level or interval. The total DQI-SNR score was aggregated into three pre-specified categories: low (0–1 points), medium (2–3 points) and high (4–6 points). It has previously been reported that participants in the MDC cohort with high compared to low DQI-SNR scores had an overall dietary pattern similar to current nutritional recommendations. [19] The observation that those with high scores were more likely to have changed their eating habits in the past (due to illness or other reasons), or to have been classified as potential energy misreporters, also prompted the exclusion of these individuals from the current study (see above). [19].

### Other Variables

Information on age and gender was obtained through each individual’s civil registration number, and lifestyle and socioeconomic information was obtained using a self-administered questionnaire. [27] Smoking was defined as never, former, or current smokers. Leisure time physical activity was assessed using questionnaire items adapted from the Minnesota Leisure Time Physical Activity Questionnaire. [28], [29] Subjects were ranked based on their leisure time physical activity score, and gender-specific tertiles created (i.e., low, moderate and high physical activity). Education was defined by the number of years of education completed or degree of educational level attained, i.e., less than nine years, nine years, high-school, or university/college degree. Cohabitation status was defined as living alone or cohabiting. Alcohol consumption was classified as zero, low, moderate or high. Participants reporting no alcohol intake in the 7-day menu book and no alcohol intake during the preceding year in the questionnaire were classified as zero alcohol consumers. For all other participants low, medium, and high alcohol consumption levels were set at alcohol intakes of <15, 15–30, and >30 g/day for women, and <20, 20–40, and >40 g/day for men. Waist circumference (cm) was measured midway between the lowest rib margin and the iliac crest; and hip circumference horizontally at the level of the greatest lateral extension of the hips. Blood pressure (mmHg) was measured by trained nurses at one of the visits to the MDC center, in the supine position after 10 min rest. Hypertension was defined as blood pressure ≥140/90 mmHg and/or current use of pressure-lowering medication. Dyslipidemia was defined as being treated with lipid-lowering medication.

### Classification of Cardiovascular Events

All subjects were followed from the baseline examination until their first episode of CV event, death, emigration from Sweden, or until December 31^st^ 2008. End-point information was obtained from the Swedish Hospital Discharge Register, the National Myocardial Infarction Register, the Stroke Register of Malmö (STROMA), and the National Cause of Death Register. [30], [31] Outcomes were coded in accordance with the 9^th^ and 10^th^ versions of the International Classification of Diseases (ICD). A CV event was defined as fatal or non-fatal myocardial infarction (MI) (ICD-9 codes: 410A-410X; ICD-10: I21), fatal or non-fatal ischemic (ICD-9, code: 434; ICD-10: I63) or non-specific stroke (ICD-9, code: 436; ICD-10: I64), or death attributable to ischemic heart disease (ICD-9 codes: 410–414; ICD-10: I20-I25), whichever came first. Ischemic stroke was diagnosed when computed tomography, magnetic resonance imaging or autopsy could verify the infarction and/or exclude hemorrhage and non-vascular disease. If neither imaging nor autopsy was performed, the stroke was classified as unspecified (ICD-9 code 436; ICD-10: I64). By definition, patients with transient ischemic attacks (ICD-9, code 435) is not included in the current definition of stroke and therefore excluded. In order to identify individuals who moved out of the city after the screening examination, we also used the National Hospital Discharge Register and the Swedish Cause of Death Register, using the same diagnosis validation procedures as for STROMA. Only subjects with ischemic stroke and MI were included in the analysis. Atherosclerosis is the major cause of acute MI, stroke and peripheral artery disease. Since different subtypes of stroke may be caused by other pathobiological mechanisms other than arterial atherosclerosis, the risk factors for hemorrhagic stroke differ from those for MI. [32] During follow-up a total of 1797 first CV events occurred; 703 in women and 1093 in men.

### Statistical Analysis

All analyses were sex-specific due to gender differences in eating patterns. Baseline differences among men and women across categories of index score were examined using ANOVA for continuous variables and χ^2^-test for categorical variables. Kaplan–Meier survival curves estimated the probability of remaining free of CV events during the follow-up period for the low, medium, and high categories of the DQI-SNR score. Hazard ratios (HR) and 95% confidence intervals (CI) for incident CV event were estimated using Cox proportional hazard regression with person-years of follow-up as the underlying time metric and the lowest DQI-SNR score category as the reference. The association between the six index components (SFAs, PUFAs, fish and shellfish, dietary fiber, fruit and vegetables and sucrose) and the risk of incident CV events were examined separately. The basic model included adjustment for age, total energy intake, method version, and season of data collection. An additional multivariate model was formulated adjusting for confounders identified from the literature, such as smoking, leisure-time physical activity, alcohol consumption, and waist circumference. Waist circumference and total energy were log-transformed to normalize data distribution. When investigating the individual components of the index the multivariate model also included mutual adjustment for the other components. Education and cohabiting status showed high-co-linearity with smoking, physical activity and alcohol consumption. Also, education, cohabitation status, hypertension, and use of lipid-lowering medication or thrombocyte aggregation inhibitor influenced the CVD risk estimate by less than 10% and were not included in the final multivariate model. In addition, to assess the sensitivity of the DQI-SNR score and incident CV events association, we also calculated HR and 95% CIs as in the full cohort (n = 28 098) (i.e., without exclusions). PASW Statistics (SPSS Statistics, version 18, 2009) and Stata 10.1 (Statistic/Data Analysis, 2007, USA) for Windows were used for all statistical calculations. All tests were two-sided and *P*-values <0.05 were considered statistically significant.

## Results

### Dietary, Lifestyle and Other Variables

Baseline differences across categories of index score are shown in Table 1. Apart from men with high diet quality being more likely to use lipid-lowering drugs and women with high diet quality having a marginally higher diastolic blood pressure, no differences in systolic or diastolic blood pressure, hypertension, or the use of statins or thrombocyte aggregation inhibitors, were observed across index score categories. Individuals with higher index scores were more likely to be older, more physically active during leisure-time, non-smokers, have a higher educational level and socioeconomic status, and less likely to live alone.

**Table 1:** Baseline characteristics of men (n = 6940) and women (n = 10186) of the Malmö Diet and Cancer cohort (1991–1996) by categories of total index score^1^.

		Men				Women				
	Low	Medium	High	*P* value*	Low	Medium		High		*P* value*
Number of subjects	1070	4356	1514		1753	5674		2758		
	Mean (standard deviation, SD)				Mean (standard deviation, SD)					
Age at baseline (years)	59 (7)	59 (7)	60 (7)	0.033	57 (8)		57 (8)		58 (8)	<0.001
Systolic blood pressure (mmHg)	144 (19)	143 (19)	144 (19)	0.691	139 (20)		139 (20)		139 (20)	0.574
Diastolic blood pressure (mmHg)	88 (10)	88 (10)	88 (10)	0.328	83 (10)		84 (10)		84 (9)	0.019
Waist (cm)	93 (10)	93 (10)	93 (10)	0.283	76 (10)		77 (14)		77 (10)	0.101
	n (%)				n (%)					
Smoking				<0.001						<0.001
Never	283 (26.5)	1241 (28.5)	533 (35.2)		708 (40.4)		2484 (43.8)		1391 (50.4)	
Former	366 (34.2)	1747 (40.1)	688 (45.5)		403 (23.0)		1515 (26.7)		823 (29.8)	
Current	420 (39.3)	1356 (31.4)	292 (19.3)		641 (36.6)		1674 (29.5)		544 (19.7)	
Living alone	237 (22.1)	711 (16.3)	218 (14.4)	<0.001	501 (28.6)		1532 (27.0)		662 (24.0)	<0.001
Educational level				<0.001						<0.001
< nine years	569 (53.4)	1903 (43.8)	591 (39.1)		745 (42.6)		2170 (38.3)		921 (33.5)	
Nine years	191 (17.9)	899 (20.8)	283 (18.6)		533 (30.5)		1787 (31.6)		895 (32.5)	
High school degree	187 (17.5)	961 (22.1)	381 (25.2)		259 (14.8)		837 (14.8)		483 (17.5)	
University/college degree	119 (11.2)	582 (13.4)	257 (17.0)		213 (12.2)		867 (15.3)		454 (16.5)	
Alcohol consumption				<0.001						<0.001
Zero	62 (5.8)	134 (3.1)	49 (3.2)		145 (8.3)		317 (5.6)		127 (4.6)	
Low	760 (71.0)	2755 (63.2)	1004 (66.3)		1342 (76.6)		4235 (74.6)		2067 (74.9)	
Medium	191 (17.9)	1074 (24.7)	357 (23.6)		229 (13.1)		957 (16.9)		493 (17.9)	
High	57 (5.3)	393 (9.0)	104 (6.9)		37 (2.1)		165 (2.9)		72 (2.6)	
Leisure-time physical activity				<0.001						<0.001
Low	447 (42.2)	1479 (34.2)	371 (24.6)		705 (40.4)		1947 (34.6)		720 (26.2)	
Medium	315 (29.7)	1460 (33.7)	525 (34.8)		553 (31.7)		1870 (33.2)		955 (34.8)	
High	298 (28.1)	1391 (32.7)	612 (40.6)		487 (27.9)		1817 (32.3)		1072 (39.0)	
Hypertension	722 (67.5)	2840 (65.2)	1031 (68.2)	0.07	942 (53.8)		3137 (55.3)		1518 (55.2)	0.52
Use of lipid-lowering drugs	8 (0.7)	68 (1.6)	30 (2.0)	0.04	8 (0.5)		42 (0.7)		20 (0.7)	0.436
Use of statins	5 (0.5)	44 (1.0)	16 (1.1)	0.22	6 (0.3)		26 (0.5)		16 (0.6)	0.512
Use of thrombocyte aggregation inhibitor	17(1.6)	87 (2.0)	21 (1.4)	0.14	13 (0.7)		57 (1.0)		25 (0.9)	0.599
Use of hormone replacement therapy	–	–	–		340 (19.9)		1151 (21.2)		570 (22.5)	0.139

1 Total index score ranged from 0 to 6 points based on adherence to six dietary components. Low score was defined as 0–1, medium score as 2–3 and high score as 4–6 points.

* ANOVA was used to calculate *P* values across categories of score for continuous variables and χ^2^-test to calculate *P* values for distribution of categorical variables across categories of score.

### DQI-SNR and Risk of Incident CVD

**Figure 1:**
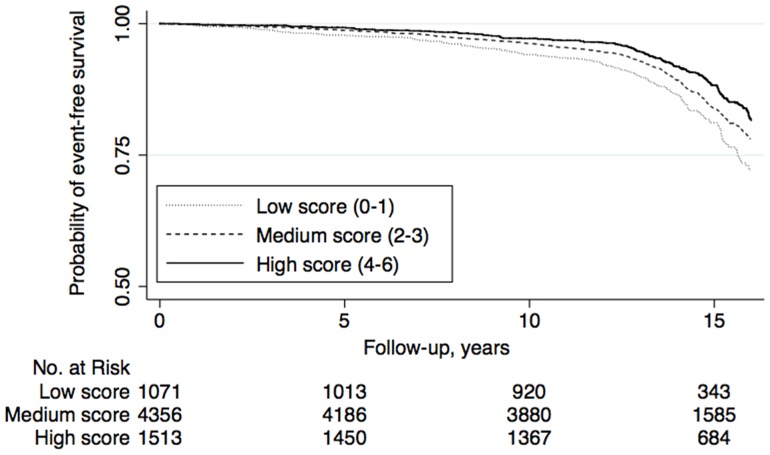
Kaplan-Meier curves of cardiovascular event-free survival by categories of DQI-SNR score among men (n = 6 940) in the Malmö Diet and Cancer cohort (1991–2008). Analysis time was cut-off at 16 years of follow-up.

**Figure 2:**
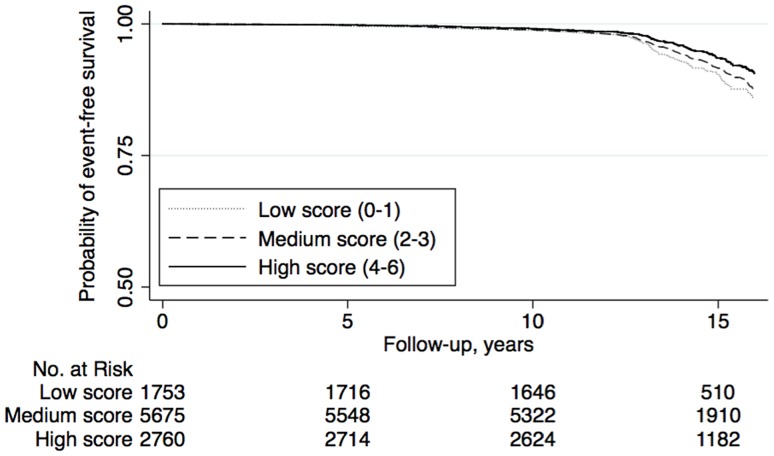
Kaplan-Meier curves of cardiovascular event-free survival by categories of DQI-SNR score among women (n = 10 186) in the Malmö Diet and Cancer cohort (1991–2008). Analysis time was cut-off at 16 years of follow-up.

Survival curves for the three categories of DQI-SNR score are shown in Figure 1 for men and Figure 2 for women. Significant trends of decreased risk were seen across the DQI-SNR score categories for both men and women (Table 2). Inclusion of waist circumference, smoking, leisure time physical activity, and alcohol consumption in the analysis did not substantially change the associations (Table 2). When examining the full cohort (i.e., without exclusions), both men and women with high scores had decreased risk of CV events, compared to those with low scores (data not shown), but these risk estimates were weaker (especially in women), compared to the main analysis.

**Table 2:** Hazard ratio (HR) and 95% confidence intervals (CI) for incident cardiovascular (CV) events^1^ in the men (n = 6940) and women (n = 10 186) of the Malmö Diet and Cancer cohort (1991–2008) by categories of total index score^2^.

	Low	Medium	High	*P*_trend_
Index score	0–1	2–3	4–6	
**Men**				
Number of cases	196	687	210	
Number of cases per 1000 person-years of follow-up	13.8	11.5	9.9	
Adjusted HR (95% CI)*	1.00 (ref)	0.81 (0.69, 0.94)	0.60 (0.49, 0.73)	<0.001
Fully adjusted HR (95% CI)†	1.00 (ref)	0.85 (0.72, 1.00)	0.68 (0.56, 0.83)	<0.001
**Women**				
Number of cases	132	394	177	
Number of cases per 1000 person-years of follow-up	5.5	5	4.5	
Adjusted HR (95% CI)*	1.00 (ref)	0.86 (0.71, 1.03)	0.66 (0.53, 0.81)	<0.001
Fully adjusted HR (95% CI)†	1.00 (ref)	0.91 (0.75, 1.10)	0.73 (0.59, 0.91)	0.006

1 Incident CV event was defined as non-fatal or fatal myocardial infarction or ischemic stroke, or death from ischemic heart disease.

2 Total index score ranged from 0 to 6 points based on adherence to six dietary components. Low score was defined as 0–1, medium score as 2–3 and high score as 4–6 points.

* HRs and 95% CIs were estimated using a Cox proportional hazards regression model adjusting for dietary assessment method version, age, total energy, and season of data collection.

† Additional adjustment for waist circumference, smoking status, leisure-time physical activity, and alcohol consumption.

### Index Components and Risk of Incident CV Events

Recommended intakes of dietary fiber (2.4–3.6 g/MJ), and fruit and vegetables (>400 g/day) were associated with decreased risk of CV events in both men and women in models adjusting for several lifestyle confounders (Table 3). However, in the mutually adjusted multivariate models, the associations remained significant only in men (Table 3). Also, recommended intakes of fish and shellfish (≥300 g/week) and of sucrose (<10 E %) were associated with a decreased risk of CV events among men (Table 3).

**Table 3:** Hazard ratios (HR) and 95% confidence intervals (CI) for incident cardiovascular (CV) events^1^ by adherence to recommended intake of the index components^2^ in men (n = 6940) and women (n = 10 186) of the Malmö Diet and Cancer study (1991–2008).

	Non-adherence	Adherence	
		Men	Women
**Saturated fatty acids**	≥14 E%	≤14 E%	≤14 E%
Subjects (men/women), %	82.4/82.8	17.6	17.2
Basic model*	1.00	0.95 (0.81, 1.11)	0.93 (0.76, 1.13)
Multivariate model†	1.00	0.99 (0.85, 1.16)	0.96 (0.78,1.16)
Mutually adjusted multivariate model§	1.00	1.06 (0.90, 1.25)	1.01 (0.82, 1.25)
**Polyunsaturated fatty acids**	<5 E % or >10E%	5–10 E%	5–10 E%
Subjects (men/women), %	21.1/27.9	78.9	72.1
Basic model*	1.00	0.97 (0.84, 1.12)	0.95 (0.80, 1.11)
Multivariate model†	1.00	0.96 (0.83, 1.11)	0.93 (0.79, 1.10)
Mutually adjusted multivariate model§	1.00	0.97 (0.84, 1.13)	0.93 (0.79, 1.10)
**Fish and shellfish**	≤300 g/week	≥300 g/week	≥300 g/week
Subjects (men/women), %	49.7/56.2	50.3	43.8
Basic model*	1.00	0.83 (0.74, 0.94)	0.93 (0.80, 1.08)
Multivariate model†	1.00	0.85 (0.75, 0.96)	0.98 (0.84, 1.15)
Mutually adjusted multivariate model§	1.00	0.87 (0.77, 0.98)	0.97 (0.83,1.13)
**Dietary fiber**	≤2.4 or >3.6 g/MJ	2.4–3.6 g/MJ	2.4–3.6 g/MJ
Subjects (men/women), %	81.1/67.6	18.9	32.4
Basic model*	1.00	0.68 (0.58, 0.80)	0.76 (0.65, 0.89)
Multivariate model†	1.00	0.77 (0.66, 0.91)	0.82 (0.70, 0.97)
Mutually adjusted multivariate model§	1.00	0.83 (0.69, 0.99)	0.87 (0.72, 1.05)
**Fruit and vegetables**	≤400 g/day	≥400 g/day	≥400 g/day
Subjects (men/women), %	71.8/59.9	28.2	40.1
Basic model*	1.00	0.74 (0.64, 0.85)	0.76 (0.65, 0.90)
Multivariate model†	1.00	0.80 (0.69, 0.92)	0.83 (0.71, 0.98)
Mutually adjusted multivariate model§	1.00	0.85 (0.73, 0.99)	0.87 (0.73, 1.04)
**Sucrose**	≥10E%	≤10E%	≤10E%
Subjects (men/women), %	27.5/32.8	72.5	67.2
Basic model*	1.00	0.80 (0.71, 0.91)	0.90 (0.77, 1.05)
Multivariate model†	1.00	0.83 (0.73, 0.95)	0.89 (0.76, 1.05)
Mutually adjusted multivariate model§	1.00	0.86 (0.75, 0.98)	0.91 (0.77, 1.07)

1 Incident CV event was defined as non-fatal or fatal myocardial infarction or ischemic stroke, or death from ischemic heart disease.

2 Adherence to the index components was dichotomized based on pre-specified recommended intake levels (adherence = 1 and non-adherence = 0) and non-adherence was used as the reference category.

* HRs and 95% CIs estimated using a Cox proportional hazards regression model adjusting for dietary assessment method version, age, total energy, and season of data collection.

† Additional adjustment for waist circumference, smoking status, leisure-time physical activity, and alcohol consumption.

§ Multivariate model (as above) with mutual adjustment for the index components in this table.

## Discussion

This study demonstrates the relationship between DQI-SNR, an index score based on the current Swedish dietary recommendations, and incident CV events in apparently healthy, middle-aged subjects. The main finding is that men and women reporting high overall adherence to current dietary recommendations had a lower risk of CV events compared to those with low adherence. A reduced risk of CV events were also seen among men who reported recommended intake levels of fish and shellfish, dietary fiber, fruit and vegetables and sucrose.

Dietary recommendations are designed to promote good overall health and reduce the risk of major chronic disease in the general population. Overall the risk reduction of CV events seen with a high diet quality in this study is in line with previous reports from U.S. and European cohorts. In a European study based on five different cohorts, high scores of a healthy diet indicator, based on the World Health Organization’s guidelines, was associated with 18% lower risk of mortality due to CVD. [18] Studies using the Healthy Eating Index (HEI), a score measuring how well Americans conform to the Dietary Guidelines for Americans and the food guide pyramid, found a reduction in CVD risk in two separate US cohorts of men (28%) and women (14%). [33], [34] Two other studies evaluating recommended diets, using the Alternate Healthy Eating Index (AHEI) based on foods and nutrients predictive of chronic disease risk, reported a reduction in CVD risk in both men and women. [14], [35] Pooled analysis of these US cohorts showed reduced risk of CVD with high scores on the HEI-2005 (21%) and the AHEI-2010 (24%). [15].

In a previous study from the MDC cohort, high DQI-SNR scores were associated with approximately 40% decreased risk of mortality due to CVD in men, but no association with CVD mortality was seen among women. [20] In contrast, this study suggests that high DQI-SNR scores are associated with reduced number of CV events in both men and women. The individual index components were more strongly associated with CV events among men in this study. Since there were fewer CV events among women compared to men in this study population, there is a possibility that the non-significant findings among women are due to reduced statistical power in the multivariate model. Further, there are possible gender differences in food selection and unobserved confounders across food patterns.

The weaker associations seen with the individual index components compared to that of using the total score as a measure of diet quality suggest the importance of considering the overall dietary pattern when investigating the associations between diet and disease. Since nutrient intakes are highly correlated, it is difficult to isolate the effect of individual nutrients in epidemiological studies. In line with a previous report based on MDC data on macronutrient intakes [36], we found no association between recommended intake levels of SFA and PUFA and risk of incident CV events in this study. However, since very few fulfilled the SNR for SFA, the potential benefit of low SFA intake is difficult (or virtually impossible) to determine in this population. In contrast, a pooled analysis of 11 cohort studies indicated that replacing SFAs with PUFAs was associated with a reduction in the risk of CHD. [37].

The main advantages of this study are the dietary data of high relative validity, large study population with virtually no loss to follow-up (<0.7%) and CV endpoints of very high relative validity. [38] Few epidemiological studies have access to similar dietary data. [39] Further, the access to an extensive data set, containing information on many potential confounders, is an important advantage. For instance, subjects reporting previous changes in eating habits, and those identified as potential misreporters of energy intake were excluded from the analysis. Few other studies have the ability to consider the possibility of bias due to these factors. The importance of these factors was demonstrated in sensitivity analysis indicating stronger associations (compared to the full cohort) when subjects with prevalent CVD and diabetes, energy misreporting, and past food habit change were excluded from analysis (data not shown).

However, some limitations need to be considered. This study consists of middle-aged to elderly men and women and therefore the generalizability of our findings to other age groups is uncertain. Another potential limitation is that only 40% of the eligible population was included in the study sample, which potentially resulted in selection bias. However, previous reports from the MDC cohort indicated that the socio-demographic structure, prevalence of obesity and smoking habits were similar compared to a mailed health survey in the same population (where 74.6% participated), while the proportion reporting good health was higher. [40] We have previously observed that few participants of the MDC cohort follow the dietary recommendations^19^ and national data suggest that compliance is generally low in Sweden. [41] Overall, only 2% of the MDC cohort followed the recommended intake level for SFAs (≤10 E %) and only 20–30% the recommended intake of fiber. [19] As a consequence, the associations observed in this study may be severely deflated. The Nordic Nutrition Recommendations, on which the SNR are based upon, are currently being revised and therefore an evaluation of the new recommendations may be needed when they are published. Finally, several lifestyle factors play an important role in the development of CVD and these may confound the associations observed in this study. Although we controlled for these factors in our analysis, the possibility of residual confounding cannot be excluded.

### Conclusion

The results suggest that a high diet quality is associated with a decreased risk of CV events. This study thus supports the effectiveness of the dietary recommendations in reducing risk of CVD in the general population, and illustrates that a combination of dietary factors needs to be considered in order to fully capture the influence of diet on disease.

## References

[1] Jacobs DR Jr, Tapsell LC (2007) Food, not nutrients, is the fundamental unit in nutrition. Nutr Rev 65(10): 439–450. 17972438 10.1111/j.1753-4887.2007.tb00269.x

[2] Mente A, de Koning L, Shannon HS, Anand SS (2009) A systematic review of the evidence supporting a causal link between dietary factors and coronary heart disease. Arch Intern Med 169(7): 659–669. 19364995 10.1001/archinternmed.2009.38

[3] Fung TT, Rexrode KM, Mantzoros CS, Manson JE, Willett WC, et al (2009) Mediterranean diet and incidence of and mortality from coronary heart disease and stroke in women. Circulation 119(8): 1093–1100. 19221219 10.1161/CIRCULATIONAHA.108.816736 PMC2724471

[4] Sofi F, Cesari F, Abbate R, Gensini GF, Casini A (2008) Adherence to Mediterranean diet and health status: meta-analysis. BMJ 337: a1344 doi: 10.1136/bmj.a1344 18786971 10.1136/bmj.a1344 PMC2533524

[5] Elmer PJ, Obarzanek E, Vollmer WM, Simons-Morton D, Stevens VJ, et al (2006) PREMIER Collaborative Research Group. Effects of comprehensive lifestyle modification on diet, weight, physical fitness, and blood pressure control: 18-month results of a randomized trial. Ann Intern Med 144(7): 485–495. 16585662 10.7326/0003-4819-144-7-200604040-00007

[6] Lien LF, Brown AJ, Ard JD, Loria C, Erlinger TP, et al (2007) Effects of PREMIER lifestyle modifications on participants with and without the metabolic syndrome. Hypertension 50(4): 609–616. 17698724 10.1161/HYPERTENSIONAHA.107.089458

[7] Blumenthal JA, Babyak MA, Hinderliter A, Watkins LL, Craighead L, et al (2010) Effects of the DASH diet alone and in combination with exercise and weight loss on blood pressure and cardiovascular biomarkers in men and women with high blood pressure: the ENCORE study. Arch Intern Med 170(2): 126–135. 20101007 10.1001/archinternmed.2009.470 PMC3633078

[8] Adamsson V, Reumark A, Fredriksson IB, Hammarström E, Vessby B, et al (2011) Effects of a healthy Nordic diet on cardiovascular risk factors in hypercholesterolaemic subjects: a randomized controlled trial (NORDIET). J Intern Med 269(2): 150–159. 20964740 10.1111/j.1365-2796.2010.02290.x

[9] Appel LJ, Moore TJ, Obarzanek E, Vollmer WM, Svetkey LP, et al (1997) A clinical trial of the effects of dietary patterns on blood pressure. DASH Collaborative Research Group. N Engl J Med 336(16): 1117–1124. 9099655 10.1056/NEJM199704173361601

[10] de Lorgeril M, Salen P, Martin JL, Monjaud I, Delaye J, et al (1999) Mediterranean diet, traditional risk factors, and the rate of cardiovascular complications after myocardial infarction: final report of the Lyon Diet Heart Study. Circulation 99(6): 779–785. 9989963 10.1161/01.cir.99.6.779

[11] Howard BV, Van Horn L, Hsia J, Manson JE, Stefanick ML, et al (2006) Low-fat dietary pattern and risk of cardiovascular disease: the Women’s Health Initiative Randomized Controlled Dietary Modification Trial. JAMA 295(6): 655–666. 16467234 10.1001/jama.295.6.655

[12] Yngve A, Hambraeus L, Lissner L, Serra Majem L, Vaz de Almeida MD, et al (2006) The Women’s Health Initiative. What is on trial: nutrition and chronic disease? Or misinterpreted science, media havoc and the sound of silence from peers? Public Health Nutr 9(2): 269–272. 16571183 10.1079/phn2006952

[13] Waijers P, Feskens E, Ocké M (2007) A critical review of predefined diet quality scores. Br J Nutr 97: 219–231. 17298689 10.1017/S0007114507250421

[14] McCullough ML, Feskanich D, Stampfer MJ, Giovannucci EL, Rimm EB, et al (2002) Diet quality and major chronic disease risk in men and women: moving toward improved dietary guidance. Am J Clin Nutr 76(6): 1261–1271. 12450892 10.1093/ajcn/76.6.1261

[15] Chiuve SE, Fung TT, Rimm EB, Hu FB, McCullough ML, et al (2012) Alternative dietary indices both strongly predict risk of chronic disease. J Nutr 142(6): 1009–1018. 22513989 10.3945/jn.111.157222 PMC3738221

[16] Belin RJ, Greenland P, Allison M, Martin L, Shikany JM, et al (2011) Diet quality and the risk of cardiovascular disease: the Women’s Health Initiative (WHI). Am J Clin Nutr 96(1): 49–57. 10.3945/ajcn.110.011221 PMC3127501 21613562

[17] Kant AK, Schatzkin A, Graubard BI, Schairer C (2000) A prospective study of diet quality and mortality in women. JAMA 283(16): 2109–2115. 10791502 10.1001/jama.283.16.2109

[18] Huijbregts P, Feskens E, Räsänen L, Fidanza F, Nissinen A, et al (1997) Dietary pattern and 20 year mortality in elderly men in Finland, Italy, and The Netherlands: longitudinal cohort study. BMJ 315(7099): 13–17. 9233319 10.1136/bmj.315.7099.13 PMC2127011

[19] Drake I, Gullberg B, Ericson U, Sonestedt E, Nilsson J, et al (2011) Development of a diet quality index assessing adherence to the Swedish nutrition recommendations and dietary guidelines in the Malmö Diet and Cancer cohort. Public Health Nutr 14(5): 835–845. 21299917 10.1017/S1368980010003848

[20] Drake I, Gullberg B, Sonestedt E, Wallstrom P, Persson M, et al (2013) Scoring models of a diet quality index and the predictive capability of mortality in a population-based cohort of Swedish men and women. Public Health Nutr 16(3): 468–478. 22643161 10.1017/S1368980012002789 PMC10271633

[21] Berglund G, Elmståhl S, Janzon L, Larsson SA (1993) The Malmö Diet and Cancer Study: design and feasibility. J Intern Med 233: 45–51. 8429286 10.1111/j.1365-2796.1993.tb00647.x

[22] Mattisson I, Wirfält E, Aronsson CA, Wallström P, Sonestedt E, et al (2005) Misreporting of energy: prevalence, characteristics of misreporters and influence on observed risk estimates in the Malmö Diet and Cancer cohort. Br J Nutr 94: 832–842. 16277789 10.1079/bjn20051573

[23] Sonestedt E, Wirfält E, Gullberg B, Berglund G (2005) Past food habit change is related to obesity, lifestyle and socio-economic factors in the Malmo Diet and Cancer Cohort. Public Health Nutr 8(7): 876–885. 16277804 10.1079/phn2005736

[24] Elmståhl S, Gullberg B, Riboli E, Saracci R, Lindgarde F (1996) The Malmö Food Study: the reproducibility of a novel diet history method and an extensive food frequency questionnaire. Eur J Clin Nutr 50: 134–142. 8654326

[25] Riboli E, Elmståhl S, Saracci R, Gullberg B, Lindgarde F (1997) The Malmö Food Study: validity of two dietary assessment methods for measuring nutrient intake. Int J Epidemiol 26: 161–173. 10.1093/ije/26.suppl_1.s161 9126544

[26] Wirfält E, Mattisson I, Johansson U, Gullberg B, Wallström P, et al (2002) A methodological report from the Malmö Diet and Cancer study: development and evaluation of altered routines in dietary data processing. Nutr J 19 1: 3. 10.1186/1475-2891-1-3 PMC149436 12537595

[27] Manjer J, Elmståhl S, Janzon L, Berglund G (2002) Invitation to a population-based cohort study: differences between subjects recruited using various strategies. Scand J Public Health 30(2): 103–112. 12028859 10.1177/14034948020300020401

[28] Taylor HL, Jacobs DR Jr, Schucker B, Knudsen J, Leon AS, et al (1978) A questionnaire for the assessment of leisure time physical activities. J Chronic Dis 31: 741–755. 748370 10.1016/0021-9681(78)90058-9

[29] Richardson MT, Leon AS, Jacobs DR Jr, Ainsworth BE, Serfass R (1994) Comprehensive evaluation of the Minnesota Leisure Time Physical Activity Questionnaire. J Clin Epidemiol 47: 271–281. 8138837 10.1016/0895-4356(94)90008-6

[30] The National board of Health and Welfare. Värdering av diagnoskvaliteten för akut hjärtinfarkt i patientregistret 1997 och 1995 (in Swedish). Stockholm, Sweden: Socialstyrelsen; 2000.

[31] Zia E, Hedblad B, Pessah-Rasmussen H, Berglund G, Janzon L, et al (2007) Blood pressure in relation to the incidence of cerebral infarction and intracerebral hemorrhage. Hypertensive hemorrhage: debated nomenclature is still relevant. Stroke 38(10): 2681–2685. 17761929 10.1161/STROKEAHA.106.479725

[32] Donnan GA, Fisher M, Macleod M, Davis SM (2008) Stroke. Lancet 371(9624): 1612–1623. 18468545 10.1016/S0140-6736(08)60694-7

[33] McCullough ML, Feskanich D, Rimm EB, Giovannucci EL, Ascherio A, et al (2000) Adherence to the Dietary Guidelines for Americans and risk of major chronic disease in men. Am J Clin Nutr 72(5): 1223–1231. 11063453 10.1093/ajcn/72.5.1223

[34] McCullough ML, Feskanich D, Stampfer MJ, Rosner BA, Hu FB, et al (2000) Adherence to the Dietary Guidelines for Americans and risk of major chronic disease in women. Am J Clin Nutr 72(5): 1214–1222. 11063452 10.1093/ajcn/72.5.1214

[35] McCullough ML, Willett WC (2006) Evaluating adherence to recommended diets in adults: the Alternate Healthy Eating Index. Public Health Nutr 9(1A): 152–157. 16512963 10.1079/phn2005938

[36] Wallström P, Sonestedt E, Hlebowicz J, Ericson U, Drake I, et al (2012) Dietary fiber and saturated fat intake associations with cardiovascular disease differ by sex in the Malmö Diet and Cancer Cohort: a prospective study. PLoS One 7(2): e31637. 22384046 10.1371/journal.pone.0031637 PMC3288044

[37] Jakobsen MU, O'Reilly EJ, Heitmann BL, Pereira MA, Bälter K, et al (2009) Major types of dietary fat and risk of coronary heart disease: a pooled analysis of 11 cohort studies. Am J Clin Nutr 89: 1425–1432. 19211817 10.3945/ajcn.2008.27124 PMC2676998

[38] Hammar N, Alfredsson L, Rosén M, Spetz CL, Kahan T, et al (2001) A national record linkage to study acute myocardial infarction incidence and case fatality in Sweden. Int J Epidemiol 30: S30–4. 11759848 10.1093/ije/30.suppl_1.s30

[39] Thiébaut AC, Kipnis V, Schatzkin A, Freedman LS (2008) The role of dietary measurement error in investigating the hypothesized link between dietary fat intake and breast cancer–a story with twists and turns. Cancer Invest 26(1): 68–73. 18181048 10.1080/07357900701527918

[40] Manjer J, Carlsson S, Elmståhl S, Gullberg B, Janzon L, et al (2001) The Malmö Diet and Cancer Study: representativity, cancer incidence and mortality in participants and non-participants. Eur J Cancer Prev 10(6): 489–499. 11916347 10.1097/00008469-200112000-00003

[41] Becker W (1999) Dietary guidelines and patterns of food and nutrient intake in Sweden. Br J Nutr 81: S113–117. 10999035 10.1017/s0007114599000951

